# An Optimization on the Neuronal Networks Based on the ADEX Biological Model in Terms of LUT-State Behaviors: Digital Design and Realization on FPGA Platforms

**DOI:** 10.3390/biology11081125

**Published:** 2022-07-27

**Authors:** Yule Wang, Osman Taylan, Abdulaziz S. Alkabaa, Ijaz Ahmad, Elsayed Tag-Eldin, Ehsan Nazemi, Mohammed Balubaid, Hanan Saud Alqabbaa

**Affiliations:** 1 School of Computer Science and Artificial Intelligence, Changzhou University, Changzhou 213164, China; 1111712014@zjut.edu.cn; 2 Department of Industrial Engineering, Faculty of Engineering, King Abdulaziz University, P.O. Box 80204, Jeddah 21589, Saudi Arabia; otaylan@kau.edu.sa (O.T.); aalkabaa@kau.edu.sa (A.S.A.); mbalubaid@kau.edu.sa (M.B.); 3 Shenzhen College of Advanced Technology, University of Chinese Academy of Sciences (UCAS), Shenzhen 518055, China; 4 Electrical Engineering Department, Faculty of Engineering and Technology, Future University in Egypt, New Cairo 11845, Egypt; 5 Imec-Vision Lab, Department of Physics, University of Antwerp, 2610 Antwerp, Belgium; 6 University Medical Services Center, King Abdulaziz University, Jeddah 21589, Saudi Arabia; halkabaa@kau.edu.sa

**Keywords:** neuron, ADEX, neuromorphic, digital FPGA implementation

## Abstract

**Simple Summary:**

The brain is an incredibly complex system possessing outstanding abilities to perform difficult tasks through a vast number of densely interconnected neurons. Aimed at discovering the underlying mechanisms of the brain, a number of spiking neural networks have been proposed to mimic biological neural dynamics. Subsequently, to perceive how the neural networks in the brain work, simulation and hardware realization of large-scale systems, similar to the brain, is an essential requirement. Behavior of a single neuron can be described by the mathematical equations in different levels of computing and biological accuracy. In this approach, a new modified ADEX model is presented based on sampling frequency by the nonlinear functions of the original model. This new model is capable for reproducing all aspects of the original model in low-error and high-degree of similarity conditions. Finally, the proposed model can be implemented on digital hardware platforms to have a real digital system. Digital results show the increase in system speed (frequency) and overall saving in hardware resources (compared by the original model and other similar works). This low-cost digital hardware is applied in large-scale neuronal networks.

**Abstract:**

Design and implementation of biological neural networks is a vital research field in the neuromorphic engineering. This paper presents LUT-based modeling of the Adaptive Exponential integrate-and-fire (ADEX) model using Nyquist frequency method. In this approach, a continuous term is converted to a discrete term by sampling factor. This new modeling is called N-LUT-ADEX (Nyquist-Look Up Table-ADEX) and is based on accurate sampling of the original ADEX model. Since in this modeling, the high-accuracy matching is achieved, it can exactly reproduce the spiking patterns, which have the same behaviors of the original neuron model. To confirm the N-LUT-ADEX neuron, the proposed model is realized on Virtex-II Field-Programmable Gate Array (FPGA) board for validating the final hardware. Hardware implementation results show the high degree of similarity between the proposed and original models. Furthermore, low-cost and high-speed attributes of our proposed neuron model will be validated. Indeed, the proposed model is capable of reproducing the spiking patterns in terms of low overhead costs and higher frequencies in comparison with the original one. The properties of the proposed model cause can make it a suitable choice for neuromorphic network implementations with reduced-cost attributes.

## 1. Introduction

The Central Nervous System (CNS) is a basic biological system which includes three vital organs: neurons, synapses, and glias [1,2]. In this network, neurons are responsible for information processing and transformation of data in different parts of the human brain. By neurons switching, the essential informations are transferred to the brain parts [3,4,5]. Neurons are high-speed organs that have high number of different connections for transferring data [6]. On the other hand, synapses are the connections between two neurons. These connections causes the coupling of two connected neurons. These coupled neurons are basic submodules of the CNS that can be realized in real states [7,8,9,10]. Furthermore, glias are the protection-based cells for neurons and regulate the coupling behaviors between different neuron connections. One of the basic forms of the glias in the brain are astrocytes, which are responsible for protecting and regulating neurons behavior [3,4,9]. From this standpoint, investigating neurons dynamic can be a vital requirements in case of neuromorphic engineering. Thus, modeling of neural dynamics and spiking neural network mechanisms have been a strong tool in analyzing and processing behavior of biological neural networks.

Interaction between these basic organs in the brain corresponds to transferring data, memory and learning [11,12,13,14]. In this approach, the important and basic block is neurons with a large number of connections. A large number of connected neurons with thousands of connection in the brain makes them vital organ in case of transferring data and informations. In this way, this important part must be investigated and analyzed to achieve an efficient system for designing real organs. These neurons have several functional roles, such as receiving, transmitting and analyzing data for producing voltage signals in all parts of the human brain [3,4,5,6]. These behaviors can be formulated by some mathematical Equations [15,16,17,18]. The basic signal behaviors of the biological neurons are modeled by these mathematical equations. In neuron modeling, two approaches are selected: first, the models which are based on the biological neurons behavior, such as Hodgkin-Huxley (HH) [2] or the ADEX neuron model [5,6,7,8], and the second is based on spike state modeling, such as Izhikevich [3,4,11,12].

Among the above list, the Izhikevich model is a computationally reasonable neuronal model that is capable of reproducing all spiking patterns. This model generates all patterns of brain spikes, which is a very significant issue in the neural networks [1,3,4]. On the other hand, the HH model is a full-biological neuron model with a high number of equations and terms and is a high-cost neuronal model [2]. It may be not acceptable for implementing in hardware form because of its high overhead costs. On the other hand, the ADEX model is a cost-effective and biological neuron model that has the both behaviors of the Izhikevich and HH models at the same time, and it is an appropriate choice in case of digital design and realization [5,6].

To realize the neuronal models in hardware state, there are different choices. To achieve a hardware implementation of the neuronal models, we have two cases: analog implementation and digital realization. For realizing in the analog case, CMOS Components are used for designing an analog approach in case of following the mechanisms of neuronal models. This process is high performance in case of speed, but long development time is its disadvantage. Moreover, two factors in digital implementation, high-silicon area and power are required, but this procedure is efficient compared by other approaches [1,2,3,4,5,6]. The advantage of digital approach is its flexible attributes, time-down in processing and power supply. Using reconfigurable digital boards, such as FPGAs cause the speed-up and flexibility in this case [8,9,10,11,12]. In the field of neural networks implementation, digital approach may be a suitable procedure [1,3,4,5,6,7,8]. Indeed, because of high-speed switching in the neuronal networks, it is required to have a accelerate hardware, which is capable of regenerating the same behavior and performance of the human neuronal cells. Different approaches are considered for implementing the neural networks in hardware state in the literature [10,11,12,13,14]. In the field of neuronal realization, since we have high-frequency networks, the digital way is a better solution. On the other hand, it is best suited for computing and digital electronics, and is less affected since noise response are analog in nature, digital hardware is flexible in implementation and digital instruments are free from observational errors such as parallax and approximation errors (compared to the analog approach). The implementation of the neuronal cells using configurable platforms such as FPGA is an attractive area in terms of low overhead costs and high-frequency digital systems [1,2,3,4,5,6,7,8,9,10]. FPGA is an integrated circuit designed to be configured by a customer or a designer after manufacturing, hence the term field-programmable. The FPGA configuration is generally specified using a Hardware Description Language (HDL), similar to that used for an Application-Specific Integrated Circuit (ASIC). Circuit diagrams were previously used to specify the configuration, but this is increasingly rare due to the advent of electronic design automation tools. FPGAs are flexible and high-speed platforms which are suitable approaches for the digital realization of neuronal networks.

Different neuronal models have been realized on FPGA hardware boards. In this approach, Yaghini Bonabi et al. [2] proposed the FPGA implementation of the HH neuronal model using different methods. Moreover, Haghiri et al. [3,4], Nazari et al. [14] and Soleimani et al. [9] presented digital FPGA realization of coupled neurons with astrocyte cells. Furthermore, Soleimani et al. [12] proposed a digital effective realization of Izhikevich neuron model in low-cost and high-frequency state. In other words, the ADEX neuron model is implemented in the literature [5,6,7,8]. In detail, Gomar et al. [5] proposed power-2-based implementation of the ADEX model without any multiplications. Furthermore, Haghiri et al. [6] presented a novel realization of the ADEX neuronal model using full-matching approximation and low-error calculations. Gomar et al. [7] also proposed another approach for implementing this model using a CPG-based method and Heidarpour et al. [8] presented a CORDIC approach for ADEX realization. In all of these papers, the authors use an approximation method to realize the final hardware. This may causes different levels of errors of two original and proposed approaches and affect the final digital hardware.

As a comparison between ADEX implementation, Gomar in [5] proposes a power-2-based function for approximating the exponential term. This conversion may cause error levels between the original and proposed models, and also based on Table 7, speed-up and overall saving are reduced. Furthermore, in this approximation, the accuracy of fraction part of modification is low and this affects on the FPGA realization. On the other hand, Gomar in [7] also implemented the ADEX model in a multiplierless state. As can be seen, in this approach, she also used the power-2-based method, which is low-accurate in case of fraction part calculation. In [6], Haghiri proposed a high-accurate approximation of the ADEX model using a power-2-based function with a high degree of similarity. In their approach, the accuracy of matching is increased, but the FPGA cost is also increased; additionally, some levels of errors between the original and proposed spiking patterns can be observed. Finally, Heidarpour in [8] presented the CORDIC algorithm for approximating the ADEX neuron model. In their way, although the accuracy will be increased, the overall saving in FPGA will be significantly decreased. This issue may affects on large number of implemented neurons on an FPGA core.

The general technique for realizing the neuron models are clarified. Capable realization of biological neuronal networks are important. In biology approach, the experimental view of neuroscience are considered to have a basic inspiration of the brain architecture. Thus, studying hardware realization of these biological-like systems can be a necessary goal. At first, the neuronal modeling can be suited. Large number of neuron models are existed for SNNs based on different dynamical mechanisms. For example, the ADEX modeling is acceptable one and capable for duplicating different patterns of spikes in the brain. In the next step, the selected model can be validated in case of timing analysis and dynamics. Since the basic (original) models have high-cost functions, it is required that modification is done to achieve a low-area and cost modeling in hardware implementation. After that, the proposed model must be validated in terms of following the all aspects of original behaviors in MATLAB software. At the second step, to evaluate the proposed approach (in hardware consideration), the FPGA boards are applied in hardware case. Indeed, the Hardware Description Language (HDL) is considered for the proposed neuronal model in ModelSim and ISE Xilinx software’s. In this case, resource utilization and costs of the original and proposed models are compared in case of digital implementation. Two basic factors in this consideration (overall saving in FPGA resources and speed-up or frequency) are compared and the validated that the proposed model is in the better state of digital realization.

This paper presents the N-LUT-ADEX (Nyquist-Look Up Table-ADEX) model, which is based on accurate sampling of the original model. Since in this model, high-accuracy matching is achieved, it can recreate a large number of spike patterns in terms of the high similarity state with the original model and also reducing the final overhead costs compared to other papers. Indeed, by accurate sampling of the nonlinear terms of the original ADEX model, the final computational error will be significantly reduced. In this approach, the Nyquist frequency converts a continuous term to a discrete one. When the bandwidth of a signal is lower than the Nyquist frequency of the sampling, the equivalent sample rate is overhead the Nyquist ratio for that certain signal. Using this procedure, the nonlinear term of the original ADEX neuron model (which is an exponential term with high-cost state for digital hardware realization) can be replaced by LUT-based memories. This model can be implemented in hardware state without using any multipliers and other nonlinear terms. In other words, these nonlinear functions are high-cost and low-frequency blocks and by replacing them with some LUT-based terms, the frequency (speed) of digital system will be increased. Then, the overhead costs are significantly reduced. Two basic factors in neural networks implementations are: large-scale approach and high-speed switching. Indeed, to achieve a real and nature-inspired neural system, these two factors must be taken into account. Consequently, using this new model, we have an efficient and modified model that can be considered for implementation in biological neural networks.

## 2. ADEX Neuron

Adaptive Exponential integrate-and-fire (ADEX) neuron is based on coupling mathematical formulation for the voltage variable and the recovery one [5,6,7,8]. This biological neuron model is described by two coupled differential equations as follows:

(1) $$C\frac{dV}{dt}=-g_{L}\left(V-E_{L}\right)+g_{L}\Delta Texp\left(f\left(V\right)\right)+I-W$$

(2) $$\tau_{\omega }\frac{dW}{dt}=a\left(V-E_{L}\right)-W$$

By the following reset equations:

(3) $$if\;V>0\;then\;\left\{\begin{array}{c} V⟵V_{r}\\ W⟵W_{r}=W+b \end{array}\right.$$

where

(4) $$f\left(V\right)=\frac{V-V_{T}}{\Delta T}$$

The first equation denotes the dynamics of membrane potential and includes an activation term with an exponential voltage dependence. Voltage is coupled to a second equation, which describes adaptation. Both variables are reset if an action potential has been triggered. The combination of adaptation and exponential voltage dependence gives rise to the name adaptive exponential integrate-and-fire model. The adaptive exponential integrate-and-fire model is capable of describing known neuronal firing patterns, e.g., adapting, bursting, delayed spike initiation, initial bursting, fast spiking and regular spiking. Different ADEX parameters are described as:

- *C*: Capacitance of the membrane voltage (pF);
-
$g_{L}$ : Leakage conductance (ns);
-
$E_{L}$ : Potential of rest in effective state (mV);
-
ΔT : Threshold factor (mV);
-
$V_{T}$ : Potential of threshold (mV);
-
$V_{r}$ : Potential of rest (mV);
-
$\tau_{\omega }$ : Time of adaptation (ms);
- *a*: Adaptation of subthreshold (ns);
- *b*: Adaptation of splike-trigger (pA);
- *I*: Stimulus current (pA).

**Figure 1:**
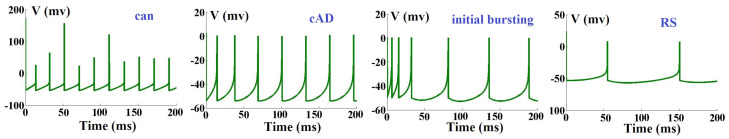
Some spiking patterns extracted by the original ADEX neuron model.

Furthermore, as depicted in Table 1, based on the variation of different ADEX parameters, we have eight spiking patterns. Based on Table 1, some spiking behaviors of the original ADEX neuron model are illustrated in Figure 1.

On the other hand, the first equation determines the membrane voltage and the injected DC current represents the input to the neuron. The variable represents the membrane potential of neuron and denotes an adaptation current variable. When the membrane voltage crosses its apex (0) , the voltage and adaptation variables will be reset according to an auxiliary. Furthermore, by scaling the parameters, the model can reproduce different types of spiking patterns, such as adapting, bursting, delayed spike initiation, initial bursting, fast spiking and regular spiking. The data presented in this study are available in Supplementary Material.

## 3. N-LUT-ADEX Modeling

To have a reduced-cost and high-speed biological neural network, the original nonlinear terms of the neuronal models must be approximated or replaced by simple and efficient terms. In this methodology, if the original high-cost model is modified to modified modeling (such the original model behaviors in all consideration), the large-scale implementation can be realized. In this paper, we have presented the N-LUT-ADEX (Nyquist-Look Up Table-ADEX) model, which is an accurate and cost-reduced approach compared to the original ADEX model. In the new approach, ADEX neuronal modeling is reformulated as below:

(5) $$C\frac{dV}{dt}=ADX\left(V\right)+I-W$$

(6) $$\tau_{\omega }\frac{dW}{dt}=a\left(V-E_{L}\right)-W$$

where

(7) $$ADX\left(V\right)=-g_{L}\left(V-E_{L}\right)+g_{L}\Delta Texp\left(f\left(V\right)\right)$$

After a continuous signal, G(t) is sampled at a constant ratio, $f_{s}$ samples. There is a continual limitless numeral of additional continuous terms that fits a similar set of samples. In terms of the terms bandwidth (B) , the Nyquist standard is regularly specified as $f_{s}>2B$ . In this method, 2B is named the Nyquist rate for functions with bandwidth B [19].

**Figure 2:**
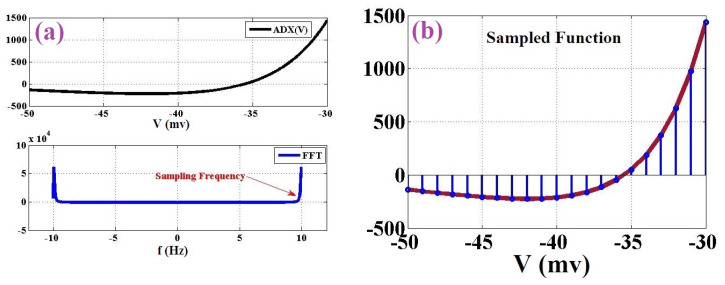
(**a**) FFT of the objective function based on the variation of voltage variable. (**b**) Sampled function based on the Nyquist rate (20 points).

In our method, as can be seen in Equation (7), the ADX(V) function is considered the objective function. At first, the Fast Fourier Transform (FFT) of this function is evaluated in case of sampling frequency. Based on the Nyquist rate, if we have the peak frequency of the function, it can be discretized to some points in the range of the voltage variable, and then, this digitized points will eventually reconstruct the objective function. The objective function ( ADX(V) ) and its FFT have been illustrated in Figure 2a. As can be seen in this figure, the base frequency of the objective function is about 10 Hz . Thus, this function is sampled by the Nyquist rate method for at least twice as much of this rate (at least 20 points of sampling). In this modification, the basic criteria for design and development of the final system are achieving a low-resources-cost, high-frequency (speed) and low-error digital design in comparison to the original basic modeling. As a result, by modifying the original ADEX neuron model, we have new high-speed, low-error and low-area hardware that can be implemented on FPGA boards as a compact digital design. In large-scale realization, this system can be considered for achieving real neural networks. These networks have the same biological behaviors as real neural networks.

**Figure 3:**
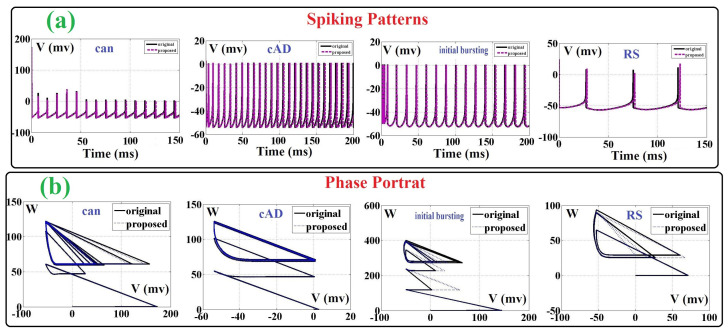
Comparison between the original and proposed ADEX in terms of spiking patterns and phase portrait. The proposed model is similar to the original ADEX model in two basic factors of the spiking patterns and phase portrait.

The final sampling result is shown in Figure 2b. As can be depicted in this figure, 20 points are selected based on the Nyquist rate. Thus, this continuous function is replaced by 20 values, and then, the nonlinearity of the ADEX neuron model can be solved. By this modification, the final overhead costs will be significantly reduced. Furthermore, the spiking shapes of two modeling (original and proposed) are compared in Figure 3a. It is showed that differences between the ADEX neuron and the proposed method is in the low-state, considerably so. For further consideration, the phase portrait of the original and proposed models are illustrated in Figure 3b. In this figure, the original and proposed models are similar at four spike patterns.

## 4. Error Methods, Dynamical Evaluation and Efficient Coefficients

Three basic issues must be evaluated for more efficient digital implementation. In this approach, first, the error level must be calculated, and second, the proposed model is evaluated in case of dynamical behaviors, and consequently, the digital coefficients have been extracted.

### 4.1. Error Methods

To validate the proposed model, the error criteria must be considered. In this way, it is important that errors level of two original and presented models are low for more efficient digital implementation. In our paper, the basic errors have been evaluated: ${\mathrm{ERR}}_{p}$ , Correlation and MAE [3,4,5,12].

-
MAE : The Mean Absolute Error (MAE) measures how far away predicted values are from observed values, and it is one of a number of ways of comparing forecasts with their eventual outcomes.
-
Correlation : Correlation is statistical relationship involving dependency between two set of spikes.
-
${\mathrm{ERR}}_{p}$ : This error is defined as the difference between the main curve (original model) and proposed model at the lowest point of the curves.

Consequently, the formulation of the error methods are given by:

(8) $$\mathrm{MAE}=\frac{1}{n}\sum_{i=1}^{n}\left|V_{Proposed}-V_{ADEX}\right|$$

(9) $$\mathrm{Corr}\left(V_{\mathrm{Proposed}},V_{\mathrm{ADEX}}\right)=\frac{cov(V_{ADEX},V_{Proposed})}{\sigma_{ADEX}\sigma_{Proposed}}$$

(10) $${\mathrm{ERR}}_{p}=\left|V_{\left(ADEX-lowest\;state\right)}-V_{\left(proposed-lowest\;state\right)}\right|$$

Error criteria are reported in Table 2. The error level calculation is in the low state in different spiking patterns. Thus, the proposed neuron model can follow the original ADEX neuron in high similarity case and reduced-error calculations. When these basic factors are in a good state, the proposed model can be realized in a digital case with a high degree of similarity and performance. In fact, to test and evaluate the presented neuron in terms of high-similarity case between spiking patterns of basic and presented modelings, these error criteria have been applied.

### 4.2. Dynamical Evaluation

To validate the proposed neuron mode, it is required that the dynamical evaluations are considered. In addition to the spiking patterns similarities, the equilibrium points and Jacobean matrix for the original and proposed models must be examined in case of points matching [3,4,5,6,7,8,9,10]. Therefore, the nullcline of the two models (original and proposed) are considered for evaluating the dynamical behaviors [19,20].

The nullcline of the original ADEX model is given by:

(11) $$\left\{\begin{array}{c} \frac{dV}{dt}=0\\ \frac{dW}{dt}=0 \end{array}\right.$$

(12) $$\left\{\begin{array}{c} W=-g_{L}\left(V-E_{L}\right)+g_{L}\Delta Texp\left(f\left(V\right)\right)+I\\ W=a(V-E_{L}) \end{array}\right.$$

and for the proposed model, this formulation can be given by:

(13) $$\left\{\begin{array}{c} W=\mathrm{ADX}(V)+I\\ W=a(V-E_{L}) \end{array}\right.$$

In this way, the Jacobean matrix is defined and can be obtained as:

(14) $$J\left(V,W\right)=\left(\begin{array}{cc} A1 & B1\\ C1 & D1 \end{array}\right)$$

where

(15) $$\left\{\begin{array}{c} A1=\frac{\partial p(V,W)}{\partial V}\\ B1=\frac{\partial p(V,W)}{\partial W}\\ C1=\frac{\partial q(V,W)}{\partial V}\\ D1=\frac{\partial q(V,W)}{\partial W} \end{array}\right.$$

In this equation, P is the derivative of the basic variables (*V* and *W*). Thus, the Jacobean matrix for the original and proposed models are presented as in Table 3. As a result test, the dynamics of two models are compared based on Figure 3b. The dynamic behavior is in a similar state.

### 4.3. Efficient Coefficients

In the case of digital realization of the biological neural networks, it is optimal that all of parameters in the proposed model are fixed to binary values. Indeed, if this condition is considered, the final digital hardware is implemented in a low-cost state. In this approach, all parameters (from Table 1) must be converted to binary values. It is mentioned that error level must be evaluated in case of low-error computations. Consequently, as can be seen in Table 4, these digital values have been presented.

All parameters have been digitized. In other words, since the goal of this paper is the digital implementation of ADEX neuron model, this helps us to convert all multiplications of the modified model to digital SHIFTs and ADDs (or SUBs), and as a result, it has a high-frequency and reduced-cost realization.

## 5. Digital Approach

**Figure 4:**
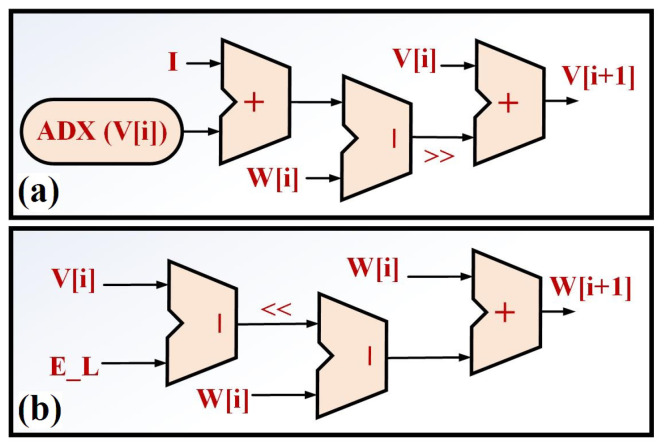
General architecture of the proposed model. (**a**) Proposed structure of the voltage variable. (**b**) Proposed structure of the recovery variable.

This section presents a multiplierless, low-cost and high-frequency design of the proposed ADEX neuron model. In this approach, since the proposed method is based on the LUT state, the final overhead costs will be reduced, significantly. The overall architectures of the proposed ADEX model are shown in Figure 4. The final hardware is composed of ADDs, SUBs and digital SHIFTs without any multipliers and other nonlinear functions. This can be an efficient and low-cost issue in comparison with the original ADEX model. The following subsections present more details.

### 5.1. Variables Discretizing

Each design consists of two blocks to calculate *V* and *W*. As the first step for implementation, it is necessary to discretize equations. In this paper, we have utilized the Euler method as:

(16) $$V\left[i+1\right]=V\left[i\right]+\left(\frac{dt}{C}\right)\left[ADX\left(V\left[i\right]\right)+I-W\left[i\right]\right]$$

(17) $$W\left[i+1\right]=W\left[i\right]+\left(\frac{dt}{\tau_{\omega }}\right)\left[a\left(V\left[i\right]-E_{L}\right)-W\left[i\right]\right]$$

By the following reset equations:

(18) $$if\;V\left[i\right]>0\;then\;\left\{\begin{array}{c} V\left[i\right]⟵V_{r}\\ W\left[i\right]⟵W_{r}=W\left[i\right]+b \end{array}\right.$$

In the above formulation, the time step of the Euler method is dt . To have a digital implementation, this value is set to dt=1/128 .

### 5.2. Bit Width Consideration

The other step is THE bit width determination of the hardware functional units to achieve minimum hardware cost. To find the bit width, we need to calculate the values’ range in each point of the hardware structures. Moreover, the maximum shifts (right and left) must be evaluated to evade any extra in bits. In the proposed ADEX model, the voltage variable variates between −60 mV to +10 mV. Thus, a bit width of 6 is required. Based on the Discretized equations, these values are shifted to right as about 20 bits. Consequently, a bit width of 27 must be calculated. Indeed, one bit is evaluated for the sign bit, and finally, a bit width of 28 is achievable.

### 5.3. Architecture View

**Figure 5:**
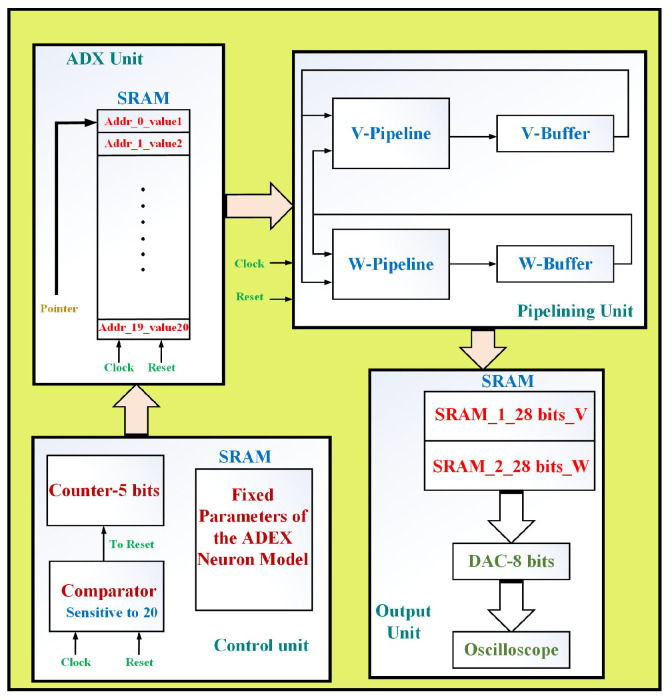
Architecture view of the proposed neuron design. This system composed of ADX unit, pipelining unit, control unit and output unit.

As mentioned, the proposed ADEX model is a multiplierless system without any nonlinear terms. In this approach, all terms and functions are realized by ADDs, SUBs and SHIFTs. The overall system architecture based on the proposed ADEX neuron is showed in Figure 5. It is illustrated that the final architecture is composed of four basic units: ADX unit, Pipelining unit, Control unit and Output unit. The ADX unit is a Static Random-Access Memory (SRAM) with 20 address points, where each address is responsible for protecting one value based on the proposed LUT. If the Clock pulse is increased, the pointer is increased and the final value of the proposed function is extracted from this unit and transferred to the pipelining unit. On the other hand, the pipelining unit is responsible for creating the final signals (*V* and *W*), which are based on the scheduling diagrams. In this unit, for producing the basic variables, two buffers have been considered which store the signals. Moreover, the control unit controls the basic signals for regulating the final system. In this way, an 5-bit counter is increased to trigger the pointer of the ADX unit. Furthermore, the comparator controls this counter for any overflow. The basic parameters of the proposed model have been stored in this unit. Finally, after storing the basic variables in the pipelining unit, these signals are transferred to output unit. In this unit, two buffers (SRAM) are available for protecting the final signals. Consequently, the basic variables are given to an DAC (8-bit Digital to Analog Converter) for generating the analog data that can be observed with an oscilloscope.

### 5.4. Minimum Resources

This part presents the minimum required resources of two basic and presented models based on the differential equations. In the presented neuron model, since all nonlinear terms, such as multiplications and exponential unit, have been removed, the final overhead costs are reduced; additionally, the maximum frequency of the system will be significantly increased. In large-scale digital implementation, these resources are very important in case of a maximum number of implemented neurons on an FPGA board. Indeed, the factor of overall saving in FPGA is responsible for increasing the number of realized neurons, and if this factor is high, a large number of ADEX neurons can be implemented on an FPGA hardware. The basic nonlinear terms in the original model are: exponential term, multipliers and dividers. These operations have high-cost realization and cause a reduction in the final frequency of the system. On the other hand, in the proposed ADEX model, these nonlinear terms have been removed, and then, the overall saving in FPGA will be significantly increased. Table 5 shows the minimum required resources of the original and proposed models.

From this table, our proposed model is of low cost with regards to the overhead costs. The proposed model only uses the digital SHIFTs and ADDs. Indeed, the proposed model is implemented on FPGA hardware without the use of multiplications, exponential units, dividers and any nonlinear functions.

### 5.5. Population View

**Figure 6:**
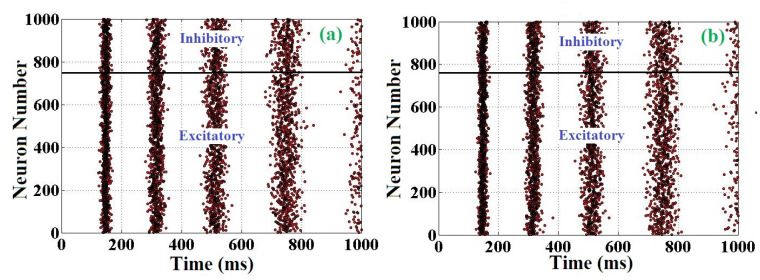
Raster plot shows the 2000 connected ADEX neurons. (**a**) Original model. (**b**) Proposed model.

**Figure 7:**
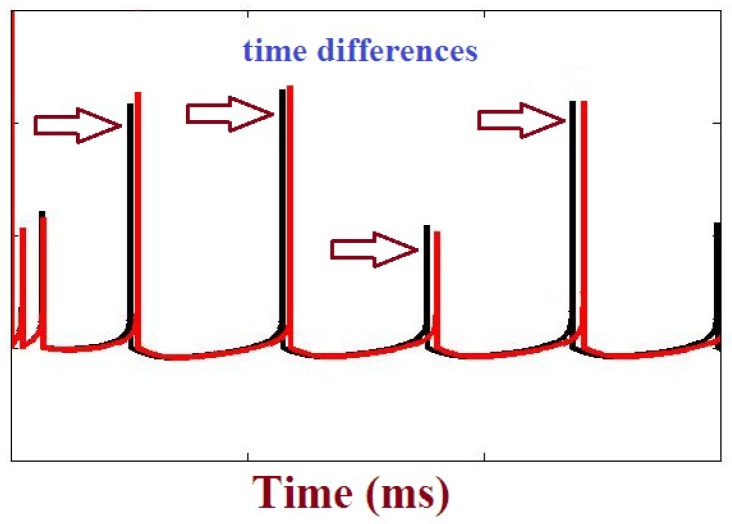
Time differences between spiking patterns of the original and proposed ADEX models.

To test and validate the proposed neuron model, a network composed of 2000 randomly connected neurons has been evaluated. This simulation is done for comparing the original and proposed ADEX models in case of population state. In this approach, a ratio of 4 to 1 is considered (excitatory to inhibitory). It is emphasized that in this population form, the connections of neurons are random. The raster plots of the original and proposed simulations are depicted in Figure 6. As can be seen, the activity of these two models is similar (same rhythm of about 6 Hz). In order to better consider the differences between the original and proposed models in network behavior, an error criterion is defined based on the Mean Relative Error (MRE). This error was applied to the proposed model, and each spike that fires at a mean value of over 1000 ms was calculated. This error can be formulated as:

(19) $${\mathrm{MRE}}_{\left(\mathrm{Proposed}\right)}\%=\frac{\sum_{i=1}^{N}\frac{|\Delta t_{\left({\mathrm{Proposed}}_{i}\right)}|}{|t_{\mathrm{si}}|}}{N}*100$$

where Δt is time difference between the $i_{th}$ spike in the proposed ADEX model and original model as depicted in Figure 7, while *N* is the number of samples. This procedure is applied for the all types of Izhikevich neuron models. Table 6 shows the MRE of 2000 randomly connected neurons for the proposed ADEX model.

## 6. Hardware Results

In this section, the proposed ADEX neuron model has been implemented on an FPGA board. The XC2VP30 FPGA board (which consists of required elements) is used for realizing the final hardware. The final hardware is synthesized and the overhead costs are presents in Table 7. As can be seen, our proposed neuron model in comparison to the original model is in a better state (frequency and costs). Moreover, the proposed model is more efficient compared to other, similar papers.

**Figure 8:**
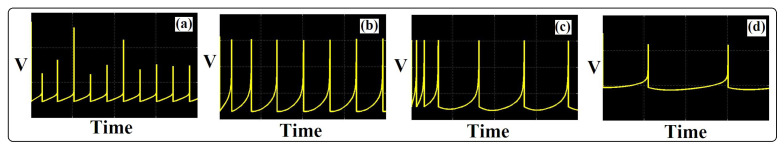
Output voltage of the proposed ADEX neuron implemented on the FPGA board. (**a**) can. (**b**) cAD. (**c**) Initial bursting. (**d**) RS.

As illustrated in this table, our proposed model provides the best results in comparison with similar papers. Two basic factors in this approach are: speed (frequency) and large-scale implementation. The proposed neuron model has high-speed behaviors, and also in the case of large-scale neuron realization, based on the overall saving factor ( $100-[{\mathrm{FPGA}}_{\mathrm{Costs}}]$ ), the saving of our proposed model is the best in comparison with other works. By this modification, using one FPGA board, more implemented neurons can be realized in hardware form. Figure 8 displays oscilloscope photographs of the proposed ADEX neuron type implementations. As can be seen, hardware signals have the same behaviors of the original simulation voltage signals in all spiking patterns. The device utilization for the implementation of about 60 neurons based on the proposed ADEX model can be realized (on an FPGA core). Consequently, our proposed neuron model is more efficient compared to the original model and other similar works in case of high-speed digital system and large-scale biological neural networks implementation.

As mentioned, the original ADEX neuron model is a nonlinear high-cost model. In this paper, the proposed model has been presented, which is capable for reproducing all patterns of the original model with a high degree of similarity. In this paper, a new ADEX modeling is applied to follow the main nervous system, that is capable to reinventing different mechanisms of the brain. The proposed approach can regenerate all mechanisms of the original ADEX neuron in high levels of performance and similarity. The new proposed case is used the Look-Up Table (LUT) modules to model the mathematical-based neurological structures, that can be implemented in high-level of equality with the original neuronal modeling. In other words, if the high-cost functions and terms are ignored, the final proposed model the will be realized and well implemented with such attributes as high-performances, low-overhead costs, and low-level computational errors. To evaluate the final digital circuit in case of validation with the original one, we have used the FPGA reconfigurable boards (Xilinx Virtex-II). After digital synthesize of digital HDL codes, it is showed that our proposed modeling is low-cost in case of FPGA resource utilization consumption and significantly follows the basic ADEX model. Finally, digital hardware results demonstrate the increasing overall saving of the presented work to 97.61% in FPGA and a higher frequency (speed-up) of the proposed model of about 212 MHz, that is higher than the basic model designing, 34 MHz. Indeed, based on these two basic factors (FPGA saving and speed-up), the proposed model is better than the original model in all aspects. Moreover, in large-scale state, the presented modeling is in the best form in comparison with the original model (in case of the maximum number of realized neurons on an FPGA board).

## 7. Conclusions

In this paper, an LUT-based modeling method of the ADEX model using Nyquist frequency method sampling is presented. This approach is a characteristic of a sampler, which converts a continuous function or signal into a discrete sequence. This new modeling is called N-LUT-ADEX (Nyquist-Look Up Table-ADEX) and is based on accurate sampling of the original ADEX model. The proposed approach exactly can follow the original ADEX model in a low error state and high degree of spiking pattern similarity. The time-domain and dynamical behaviors show that this new model is capable of reproducing all aspects of the original model. The main nonlinear terms of the original model are converted to LUT-based terms without any multipliers, dividers and exponential parts, which makes the proposed model an efficient approach. The implementation of this proposed model on an FPGA Virtex-II board shows that the new model is of low cost with high-speed attributes, which results in a real biological neural network. Two basic factors of the neuronal realization (speed-up and large-scale implementation) provide good results compared to the ADEX original neuronal model and other similar works. In this way, there is an overall saving of 97.61% ; additionally, a speed-up to 212 MHz has been achieved. To test the population network, a system composed of 2000 randomly connected neurons is simulated. The proposed model in this simulation test is is highly similar to the original ADEX neuron. Our proposed model performs better than other works; Gomar et al. [5,7], Haghiri et al. [6] and Heidarpour et al. [8] obtained overall saving of 95% , 94% , 96.7% and 70.76% , respectively. Moreover, the frequencies in these works are 187, 187.5 , 196 and 134 MHz, respectively. Among all similar works, our proposed model obtains better results. Other similar papers present ADEX neuron implementation with different approaches. In these mentioned papers, in some cases, the accuracy of realization is reduced. In some implementation, this accuracy will be increased, but the overall saving in FPGA resources is decreased. In our proposed model (N-LUT-ADEX), since the original functions are sampled (without any approximation), a high degree of accuracy is achieved. On the other hand, the overall saving in FPGA will be increased when all nonlinear terms of the original model are removed. Moreover, because all multiplications are removed, the final frequency will be significantly increased.

## Figures and Tables

**Table 1:** ADEX parameters for generating different spiking patterns.

Spiking Type	C	$g_{L}$	$E_{L}$	$V_{T}$	ΔT	a	$\tau_{\omega }$	b	$V_{r}$	I
Tonic spiking	200	10	−70	−50	2	2	30	0	−58	500
Adaptation	200	12	−70	−50	2	2	300	60	−58	500
Initial bursting	130	18	−58	−50	2	4	150	120	−50	400
Delayed accelerating	200	12	−70	−50	2	−10	300	0	−58	300
Irregular spiking	100	12	−60	−50	2	−11	130	30	−48	160
can	59	2.9	−62	−42	3	1.8	16	61	−54	184
cAD	83	1.7	−59	−56	5.5	2	41	55	−54	116
RS	104	4.3	−65	−52	0.8	−0.8	88	65	−53	98

**Table 2:** ${\mathrm{ERR}}_{p}$ , MAE and correlation computations for different spiking patterns.

Neuron Mode	${\mathrm{ERR}}_{p}$	MAE	Correlation%
Tonic spiking	0.02	0.13	98
Adaptation	0.03	0.17	95
Initial bursting	0.26	0.14	97.5
Delayed accelerating	0.21	0.21	99
Irregular spiking	0.21	0.74	91
can	0.48	0.52	96
cAD	0.41	0.21	96
RS	0.13	0.43	99

**Table 3:** The Jacobean matrix equations for two ADEX models (original and proposed).

Neuron	A1	B1	C1	D1
Original ADEX	$\frac{g_{L}}{C}\left[exp\left(f\left(V\right)\right)-1\right]$	$\frac{-1}{C}$	$\frac{a}{\tau_{\omega }}$	$\frac{-1}{\tau_{\omega }}$
Proposed ADEX	0	−1	$\frac{a}{\tau_{\omega }}$	$\frac{-1}{\tau_{\omega }}$

**Table 4:** ADEX digital parameters using in hardware implementation (based on the spiking patterns of Table 1).

C	$g_{L}$	$E_{L}$	$V_{T}$	ΔT	a	$\tau_{\omega }$	b	$V_{r}$	I
128 + 64 + 8	8 + 2	−64 − 4 − 2	−32 − 16 − 2	2	2	16 + 8 + 4	0	−32 − 16 − 8	512 − 8 − 4
128 + 64 + 8	8 + 4	−64 − 4 − 2	−32 − 16 − 2	2	2	256 + 32 + 8	64 − 4	−32 − 16 − 8	512 − 8 − 4
128 + 2	16 + 2	−32 − 16 − 8 − 2	−32 − 16 − 2	2	4	128 + 16 + 4	128 − 8	−32 − 16 − 2	256 + 128 + 16
128 + 64 + 8	8 + 4	−64 − 4 − 2	−32 − 16 − 2	2	−8 − 2	256 + 64 − 16	0	−32 − 16 − 8 − 2	256 + 64 − 16
64 + 32 + 4	8 + 4	−64 + 4	−32 − 16 − 2	2	−8 − 2 − 1	128 + 2	16 + 8 + 4	−32 − 16	128 + 32
−32 − 16 − 8	2 + 1	−64 + 2	−32 − 8 − 2	2 + 1	2−(1/4)	16	64 − 2 − 1	−32 − 16 − 4	128 + 64 − 8
64 + 16 + 4	1 + (1/2)	−64 + 4 + 1	−64 + 8	4 + 1 + (1/2)	2	32 + 8 + 1	64 − 8 − 1	−64 + 8 + 2	128 − 8 − 4
64 + 32 + 8	4 + (1/2)	−64 − 1	−64 + 8 + 4	1	− 1	64 + 16 + 8	64 + 1	−64 + 8 + 2 + 1	64 + 32 + 2

**Table 5:** Minimum required resources of the original and proposed ADEX models.

Model	Exponential	Multiplier	Divider	Adder	Subtractor
Original ADEX	1	6	5	5	6
Proposed ADEX	0	0	0	3	3

**Table 6:** Mean relative error for all spiking patterns in the proposed model.

Neuron Type	MRE %
Tonic spiking	1.21
Adaptation	0.96
Initial bursting	1.14
Delayed accelerating	2.3
Irregular spiking	2.03
can	1.17
cAD	1.16
RS	1.07

**Table 7:** Resource costs of FPGA hardware for neuron model implementation.

Reference	Slice Flip Flop	4-In-LUT	Speed	Overall Saving
Original ADEX (Virtex II)	956 (3.50%)	1753 (6.4%)	34 MHz	90.1%
Proposed ADEX (Virtex II)	185 (0.67%)	472 (1.72%)	212 MHz	97.61%
Gomar et al. [5] (Virtex II)	388 (1%)	1279 (4%)	187 MHz	95%
Gomar et al. [7] (Virtex II)	530 (1%)	1420 (5%)	187.5 MHz	94%
Haghiri et al. [6] (Virtex II)	270 (1%)	643 (2.3%)	196 MHz	96.7%
Heidarpour et al. [8] (Spartan 6)	829 (7.24%)	1221 (22%)	134 MHz	70.76%

## Data Availability

The data presented in this study are available in Supplementary Material.

## Supplementary Materials

The following supporting information can be downloaded at: https://www.mdpi.com/article/10.3390/biology11081125/s1.
