# Correction: Wang et al. An Optimization on the Neuronal Networks Based on the ADEX Biological Model in Terms of LUT-State Behaviors: Digital Design and Realization on FPGA Platforms. *Biology* 2022, *11*, 1125

**DOI:** 10.3390/biology12010052

**Published:** 2022-12-28

**Authors:** Yule Wang, Osman Taylan, Abdulaziz S. Alkabaa, Ijaz Ahmad, Elsayed Tag-Eldin, Ehsan Nazemi, Mohammed Balubaid, Hanan Saud Alqabbaa

**Affiliations:** 1 School of Computer Science and Artificial Intelligence, Changzhou University, Changzhou 213164, China; 2 Department of Industrial Engineering, Faculty of Engineering, King Abdulaziz University, P.O. Box 80204, Jeddah 21589, Saudi Arabia; 3 Shenzhen College of Advanced Technology, University of Chinese Academy of Sciences (UCAS), Shenzhen 518055, China; 4 Electrical Engineering Department, Faculty of Engineering and Technology, Future University in Egypt, New Cairo 11845, Egypt; 5 Imec-Vision Lab, Department of Physics, University of Antwerp, 2610 Antwerp, Belgium; 6 University Medical Services Center, King Abdulaziz University, Jeddah 21589, Saudi Arabia

## 1. Correction Funding

There was an error in the original publication [1]. The funding section in the original publication includes two funding statements from two different universities, which need to be split into the funding and acknowledgment sections. The original publication and corrected form are mentioned as follows:

### 1.1. Original Publication

**Funding**

This work was funded by the Deanship of Scientific Research (DSR), King Abdulaziz University, Jeddah, under grant No. (G: 405-135-1443). The authors, therefore, gratefully acknowledge the technical and financial support from the DSR. Additionally, the authors were supported by the Engineering Mathematics and Physics Department, Faculty of Engineering and Technology, Future University in Egypt, New Cairo 11845, Egypt.

### 1.2. Corrected Publication

**Funding**

This work was funded by the Deanship of Scientific Research (DSR), King Abdulaziz University, Jeddah, under grant No. (G: 405-135-1443). The authors, therefore, gratefully acknowledge the technical and financial support from the DSR. 

**Acknowledgment**

Additionally, the authors were supported by the Engineering Mathematics and Physics Department, Faculty of Engineering and Technology, Future University in Egypt, New Cairo 11845, Egypt.

The authors state that the scientific conclusions are unaffected. This correction was approved by the Academic Editor. The original publication has also been updated.
